# Roles of microRNAs in Regulating Apoptosis in the Pathogenesis of Endometriosis

**DOI:** 10.3390/life12091321

**Published:** 2022-08-26

**Authors:** Izyan Nabila Ahmad Azam, Norhazlina Abdul Wahab, Mohd Helmy Mokhtar, Mohamad Nasir Shafiee, Norfilza Mohd Mokhtar

**Affiliations:** 1 Department of Physiology, Faculty of Medicine, Universiti Kebangsaan Malaysia, Cheras, Kuala Lumpur 56000, Malaysia; 2 Department of Obstetrics & Gynecology, Faculty of Medicine, Universiti Kebangsaan Malaysia, Cheras, Kuala Lumpur 56000, Malaysia

**Keywords:** endometriosis, dysregulated, microRNA, modulation, apoptosis

## Abstract

Endometriosis is a gynecologic disorder characterized by the presence of endometrial tissues outside the uterine cavity affecting reproductive-aged women. Previous studies have shown that microRNAs and their target mRNAs are expressed differently in endometriosis, suggesting that this molecule may play a role in the development and persistence of endometriotic lesions. microRNA (miRNA), a small non-coding RNA fragment, regulates cellular functions such as cell proliferation, differentiation, and apoptosis by the post-transcriptional modulation of gene expression. In this review, we focused on the dysregulated miRNAs in women with endometriosis and their roles in the regulation of apoptosis. The dysregulated miRNAs and their target genes in this pathophysiology were highlighted. Circulating miRNAs as potential biomarkers for the diagnosis of endometriosis have also been identified. As shown by various studies, miRNAs were reported to be a potent regulator of gene expression in endometriosis; thus, identifying the dysregulated miRNAs and their target genes could help discover new therapeutic targets for treating this disease. The goal of this review is to draw attention to the functions that miRNAs play in the pathophysiology of endometriosis, particularly those that govern cell death.

## 1. Introduction

Endometriosis is a benign disorder affecting 5–10% of women of reproductive age. It is characterized by the presence of endometrial glands and stroma on ectopic locations such as the pelvic cavity, ovaries, and rectovaginal septum [1,2]. It is an estrogen-dependent chronic inflammatory condition associated with pelvic pain, dyspareunia, dysmenorrhea, and infertility [3,4]. The incidence of the disease is as high as 35–50% in the general population, with symptoms of pain and infertility. Still, the disease remains underdiagnosed, and it takes a long time for a diagnosis to be made [5]. The prevalence of endometriotic disorders peaks between 25 and 35 years of age [6,7]. However, about 62% of endometriosis cases were detected among adolescents, confirmed by laparoscopy with the manifestation of chronic pelvic pain, which occurred cyclically or acyclic, as reported by Laufer et al. [8]. Meanwhile, cancer antigen-125 (CA-125) is one of the most investigated blood biomarkers, with an adequate evaluation of the test accuracy. Although this method is minimally invasive, cost-effective, and readily available compared to surgery, the effectiveness of these biomarkers remains to be assessed, since there is still insufficient evidence to determine their efficiency. Moreover, these biomarkers do not appear to be as effective as diagnostic surgery for application in clinical settings [9]. For women with endometriosis-associated pain, medical or surgical treatment can be adopted with oral contraceptives to be the first-line treatment [10]. Pain-dependent progestin therapy is better suited for its variable efficacy, as reviewed by Abdul Karim et al. [11]. Oral progestins such as norethisterone acetate (NETA) [12], cyproterone acetate (CPA) [13], medroxyprogesterone acetate (MPA) [14], and dienogest (DNG) [15] were found to be effective in reducing pain in endometriosis and improving the quality of life. However, side effects such as bone density loss are associated with DNG [11,16]. More information on the molecular pathogenesis of endometriosis is needed. Therefore, our goal in this review is to explore the miRNAs that play a role in regulating apoptosis in endometriosis. 

### Pathogenesis of Endometriosis

The origin of endometriosis remains unclear, and it has still been a controversial topic until now. Several theories have emerged regarding the pathogenesis of endometrial cells at ectopic sites, including retrograde menstruation, endometrial stem cell implantation, celomic metaplasia, and Müllerian remnant abnormalities [4,10]. Sampson proposed the most widely accepted theory in the 1920s with the occurrence of retrograde menstruation in reproductive women. According to this theory, there is a backflow of endometrium-like tissue from the uterine cavity through the fallopian tubes and then implanted in the peritoneal cavity [17]. This reflux contributes to the accumulation of lesions in the peritoneal cavity [10]. Though the incidence of retrograde menstruation is a common physiological process as it occurs in approximately 90% of women [18,19], only 10–15% of women have endometriosis [20]. Therefore, there must be some explanation for the endometriosis that develops in this group of women.

The pathogenesis of endometriosis is multifactorial, as more evidence has shown the involvement of hormonal factors, altered immunological factors, and genetic factors that contribute to the cell survival, cell proliferation, and the formation of ectopic endometrial tissues and stroma [21]. It is suggested that an altered immune system in women with endometriosis may prevent the clearance of refluxed endometrial tissue from the peritoneal cavity [2]. The cell-mediated immunity could probably defect in these women, as leucocytes do not recognize the ectopic endometrial tissue as a foreign substance [22]. When endometriosis develops, the altered immune factors may contribute to the progression and severity of the disorder. There is an increased number of leucocytes and macrophages in the endometrial implants and peritoneal fluid. These cells secrete cytokines and growth factors such as interleukins (IL-1, IL-6, and IL-8); tumor necrosis factor (TNF); regulated upon activation, normal T-cell-secreted (RANTES); transforming growth factor β1 (TGFB1); and vascular endothelial growth factors (VEGF), which cause proliferation of the implants at the ectopic site [23,24,25,26].

Hormonal factors such as endogenous and exogenous estrogens are potential modulators for endometriosis [27]. This is in line with the incidence of the disease that predominantly affects reproductive-age women and less in the post-menopausal age [28]. In addition, molecular evidence showed that the endometriotic lesion is estrogen-dependent, as there is an increased expression of aromatase and 17β-hydroxysteroid dehydrogenase type 2 in the endometriotic tissue compared to the eutopic endometrium [29]. Other factors that facilitate the pathogenesis of endometriosis include increased chemokines and matrix metalloproteinases in peritoneal fluid, angiogenesis, tumor suppressor, and oncogenes, and the upregulation of proinflammatory cytokines [23,29,30,31,32].

Another theory is celomic metaplasia, which suggests the celomic epithelium of the ovaries and peritoneum serous membrane contains undifferentiated cells that could undergo metaplastic change into endometrial cells [33]. Multiple studies indicate that stem cells may play a role in the pathogenesis of endometriosis, because the endometrium storage of progenitor stem cells enables rapid regeneration in each menstrual cycle [34]. Bone marrow-derived stem cells may also differentiate into endometrial cells and migrate to ectopic sites such as in the peritoneal cavity, thus contributing to the development of endometriosis [35,36].

Emerging evidence of molecular defects in endometrial cells contributes to endometriotic lesions. A previous study showed a significant difference in gene expression profiles between the eutopic endometrium of endometriosis and normal endometrium [37]. Alteration in the gene expression for cell adhesion [38] and invasion [39] resulted in functional changes in the eutopic tissues and contributed to the ectopic implantations. The regulation of the genes is influenced by a small, non-coding RNA, microRNA (miRNA). Multiple human reproductive tract diseases such as preeclampsia [40], endometrial adenocarcinoma [41], uterine leiomyomata [42,43], ovarian cancer [44], and endometriosis [21,45,46,47], have documented the irregular expression of miRNA. Dysregulated miRNAs may contribute to the mechanism underlying the pathogenesis of endometriosis [46,48].

Many studies reported miRNA expression in endometriosis, but only a small group are well-characterized. Thus, there is a lack of understanding of the endometriosis pathophysiology pathway and the involvement of miRNAs in pathogenesis. In this review, we summarize the findings that evaluated the altered miRNA expression in the ectopic and eutopic endometrial tissue of women with endometriosis and the healthy controls and the potential of circulating miRNAs as biomarkers of the disease. In addition, this review also focuses on apoptosis as the pathophysiology pathway that impacted endometriosis.

## 2. microRNAs

microRNAs (miRNAs) are naturally occurring, small, non-coding RNA fragments that regulate various cellular processes, including cell proliferation, differentiation, and apoptosis [49]. A single miRNA molecule may affect different mRNA targets traversing several cellular pathways [50]. From miRNA sequences identified in 271 organisms, there are 38,589 hairpin precursors and 48,860 mature miRNAs (miRBase.org, updated March 2018, Release 22) [51,52]. These miRNAs act by suppressing gene expression post-transcriptionally by base pairing to the 3′ untranslated region (3′ UTR) of the target mRNAs [53]. microRNA is believed to act as a gene repressor by targeting mRNA cleavage and translational repression [54]. Therefore, if a miRNA is upregulated, its functional targets should be downregulated due to the increased miRNA binding to its 3′ UTRs. Alternatively, if a miRNA is downregulated, it will upregulate its functional target mRNA.

**Figure 1:**
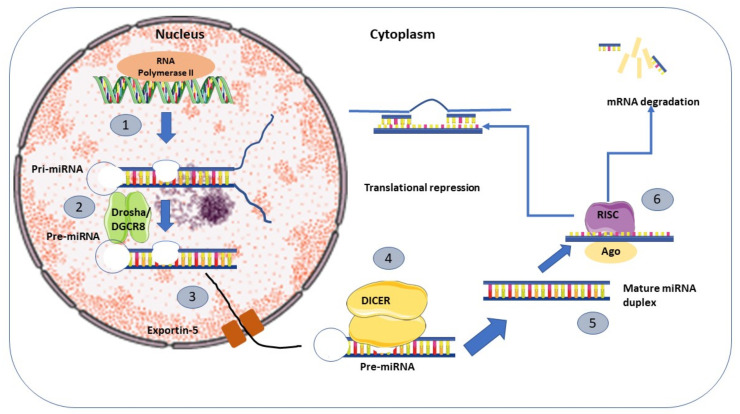
Biogenesis mechanism of microRNA. (1) Transcription of the miRNA precursor under the influence of RNA Polymerase II into primary miRNA (pri-miRNA). (2) Maturation processing of pri-miRNA utilizes nuclear cleavage by Drosha and cofactor DCGR8 into pre-miRNA. (3) The pre-miRNA is transported from the nucleus into the cytoplasm by Exportin-5. (4) The pre-miRNA in the cytoplasm undergoes further maturation by Dicer cleaving one end of the miRNA to form a mature miRNA duplex. (5) The mature miRNA duplex transforms into single-stranded transcripts by helicase. (6) The single-stranded transcript miRNA incorporated with a ribonucleoprotein complex known as the RNA-induced silencing effector complex (RISC) and guide to complementary mRNA sequences that result in mRNA degradation, translational repression, or inhibition.

The biogenesis mechanism of miRNA is shown in Figure 1. The majority of miRNAs are embedded in intronic regions of protein-encoding genes [55,56] and are transcribed in the nucleus as the primary miRNA (pri-miRNA) by RNA polymerase II [57]. Further maturation processing of pri-miRNA utilizes endonucleases, Drosha [58], and cofactor DCGR8 into 70nt hairpin pre-miRNAs [59]. The pre-miRNAs were exported into the cytoplasm through exportin-5 [60] and Dicer cleaved the pre-miRNAs into a mature miRNA-miRNA duplex [61,62], and the helicase unwound them into single-stranded transcripts. The mature single-stranded miRNAs will guide the RNA-induced silencing effector complex (RISC) to complementary mRNA sequences [46], which results in the degradation of target mRNA or the inhibition of translation. Several studies indicate that miRNAs can be transported into systemic circulation in exosomes or microvesicles, which can be incorporated into distant cells [63]. microRNAs seem to be highly stable in circulation, and their plasma profiles change markedly depending on diseases, thus having potential as disease biomarkers [64].

## 3. Dysregulated miRNAs in Endometriosis

The endometrial profiling studies of miRNAs involved in the pathogenesis of endometriosis usually compare the expression levels between all tissues. The most common method is to compare the eutopic endometrium, endometrial lining inside the uterus, and ectopic endometrium from the same patient. Still, some studies compare the eutopic endometrium of the patient with endometriosis and the healthy controls [24]. Several studies have shown that many miRNAs are involved in endometriosis, and their expression is altered in eutopic endometrium and ectopic endometrium of the same women with endometriosis [21,46]. Therefore, significant miRNAs are expected to inhibit gene expression, as proinflammatory and immune responses, angiogenesis, cell cycle progression, and adhesion molecules are expressed differently in eutopic and ectopic endometrial tissue of women with endometriosis.

Ohlsson et al., (2009) advocated that miRNA might be novel and promising candidates for diagnostic biomarkers and therapeutic targets of endometriosis [46]. microRNA arrays and RNA sequencing are the standard approaches for describing the global expression profiling of miRNAs. Ohlsson et al., (2009) also screened miRNA expression by a microarray analysis in paired ectopic and eutopic endometrial tissues and identified 14 upregulated (miR-145, miR-143, miR-99a, miR-99b, miR-126, miR-100, miR-125b, miR-150, miR-125a, miR-223, miR-194, miR-365, miR-29c, and miR-1) and 8 downregulated (miR-200a, miR-141, miR-200b, miR-142-3p, miR-424, miR-34c, miR-20a, and miR-196b) [46]. Hawkins et al., (2011) also found 10 upregulated (miR-202, miR-193a-3p, miR-29c, miR-708, miR-509-3-5p, miR-574-3p, miR-193a-5p, miR-485-3p, miR-100, and miR-720) and 12 downregulated (miR-504, miR-141, miR-429, miR-203, miR-10a, miR-200b, miR-873, miR-200c, miR-200a, miR-449b, miR-375, and miR-34c-5p) miRNAs in endometriotic lesion compared with normal endometrium [65].

### 3.1. Comparison of miRNAs Expression between Eutopic and Ectopic Endometrial Tissues

Many studies compare miRNA transcripts expressed within the ectopic tissue with paired or unpaired eutopic endometrial tissue from women with endometriosis or healthy controls by using next-generation sequencing or miRNA assays to validate the miRNAs (Table 1) [21,48,65,66,67,68,69,70,71]. However, the miRNA expression and regulation across these studies showed minimal congruence. These may occur because of different experimental designs, patient selection variability, and studies’ bioinformatics assessments.

It has been shown that 239 miRNAs are dysregulated, and nine miRNAs were differentially expressed: miR-202, miR-100, miR-29c, miR-200a, miR-200b, miR-200c, miR-25, miR-375, and miR-20a [21,48,65,66,67,68,69,70,71].

Using TARGETScan and PICTAVERT, Filigheddu et al., (2010) predicted the target mRNAs of the differentially expressed miRNAs, resulting in more than 3000 predicted targets. The functions of each predicted target were then mapped, and the molecular pathway was identified using Ingenuity Pathways Analysis (IPA 7.5) [21]. The molecular networks corresponding to biological functions associated with endometriosis (cell proliferation, cell cycle, cell motility, and reproductive system disorders) represented in IPA results were highly significant [21,24].

The molecular network converging on estrogen receptor 1 (ESR1) includes DNA methyltransferases (DNMT3A and DNMT3B) that are validated targets of miR-29b; miR-29c; and miR-29b, miR-29c, and miR-148a, respectively. These DNA methyltransferases were expressed differently in endometriosis, and there may be abnormal methylation of HOXA10 and the progesterone receptor PR-B that affects gene expression [21]. In addition, a validated target of the miR-200 family known as Smad Interacting Protein 1 (SIP1), a factor involved in epithelial-to-mesenchymal transition (EMT) and tumor metastasis [72]. In endometriotic lesions, an observed downregulation of the miR-200 family may play a role in the development of ectopic tissue.

In the Hawkins et al., (2011) study, the expression of miR-29c was upregulated, and the overexpression led to a blunted decidualization response [65]. miR-29c affects the different extracellular matrix gene expressions in the endometrial stromal cells. It has been suggested that miR-29c is a progesterone-responsive miRNA, as it is upregulated in the mid-secretory endometrium [73]. miR-34c-5p was significantly downregulated in the early secretory endometrium of women with endometriosis, and miR-193a, miR-29c, miR-203, and miR-200c were downregulated in the late proliferative endometrium [65].

Shen et al., (2013) demonstrated an increase in Steroidogenic Factor 1 (SF-1) mRNA expression in eutopic and ectopic endometrium, and this was associated with reduced levels of miR23a and miR23b. These miRNAs may function as negative feedback regulators to mediate the posttranscriptional regulation of estrogen signaling pathways, resulting in reduced SF-1 mRNA expression in the corresponding tissues [68].

### 3.2. microRNAs Expression in Eutopic Endometrial Tissue

Studies that demonstrated the altered miRNAs expression in the eutopic endometrial tissue from women with endometriosis are listed in Table 2. Six miRNAs were found to be downregulated (miR-9, miR-9*, miR-34b*, miR-34c-5p, and miR-34c-3p, and miRPlus_42 780) when eutopic endometrium from women with endometriosis and without endometriosis was compared [45]. miR-9 was the most significantly dysregulated miRNA in the microarray dataset, and qRT-PCR showed a significant reduction in women with endometriosis. One of the miR-9-predicted targets is BCL2, a gene encoding an anti-apoptosis protein and were found to have higher expression in endometriosis patients. Three members of the miR-34 family were downregulated in the endometriosis patients. The downregulation of miR-34c and miR-34b in women with endometriosis may regulate progesterone resistance and enhance proliferation and ectopic tissue survival [45]. In addition, Corney et al., (2007) concluded that miR-34b and miR-34c act as mediators of the p53-dependent suppression of proliferation [74].

Meanwhile, a study by Zhou et al., (2016) comparing miRNA expression in eutopic endometrium from women with endometriosis (*n* = 20) vs. controls (*n* = 20) revealed 54 downregulated and 12 upregulated miRNAs [79]. The overexpression of miR-196a in the eutopic endometrium inhibited the expression of the progesterone receptor and progesterone receptor B. Additionally, miR-196a upregulates MEK/ERK signaling by downregulating PGR expression in the eutopic endometrium of women with minimal or mild endometriosis [79]. The upregulation of miR-21 was found in patients with severe endometriosis after comparing miRNA expression in the eutopic endometrium from mild endometriosis and severe endometriosis [75]. Therefore, the predicted targets for miR-21 are the tumor suppressor genes PTEN, PDCD4, E2F1, and TGFBRII [80].

We observed limited concordance in the dysregulated miRNA expression identified by these studies. This could be due to the variation of clinical studies, the different locations of tissue biopsy and the experimental design, which may contribute to no or little overlap between miRNAs found in the studies. There is no overlap of miRNAs between the studies that compared the expression of miRNAs in eutopic endometrium of women with endometriosis to the controls. 

### 3.3. Circulating miRNAs as Diagnostic Biomarkers in Endometriosis

The gold standard for diagnosing endometriosis is laparoscopic visualization of endometriotic lesions with histological confirmation of the lesion [81]. However, most women who suffer from endometriosis experience a diagnostic delay of 6–12 years, because this delay is usually associated with the disease’s severity [82]. There are still challenges in proposing a reliable, noninvasive diagnostic biomarker for endometriosis, so serum/blood samples of miRNAs will reduce the surgical risk and may allow for early treatment of the disease. In addition, there is the potential for serum miRNAs to be used as biomarkers of disease progression, since there is evidence of dysregulated miRNAs in the blood of women with endometriosis [49]. Six studies in this review examined the circulating miRNAs using high-throughput assays of either serum [83,84,85] or blood/plasma samples [86,87,88] from women with or without endometriosis (Table 3). These studies showed a slight overlap of miRNAs expression across studies.

Two studies confirmed the let-7 family was dysregulated in the serum of women with endometriosis [83,88]. Let-7 promotes cell differentiation and may act as a tumor suppressor. Meanwhile, let-7b is associated with cyclin D1, an estrogen-regulated gene involved in cell proliferation [83]. Another study showed the downregulation of let-7, which targets the KRAS gene. The loss of this miRNA may initiate the progression of ectopic lesions. It may represent a link between inflammation and tumor-like growth in endometriosis [88]. Moreover, twenty-seven miRNAs were downregulated in a study comparing plasma samples from women with and without endometriosis [86]. miR-17-5p and miR-20 downregulation cause upregulated mRNA targets BCL2 and cell cycle repressor cyclin-dependent kinase inhibitor 1A, which enhances cell survival and represses cell proliferation [86]. The downregulation of miR-17-5p/20a is also associated with the upregulation of hypoxia-inducible transcription factors (HIF-1a) [89] and vascular endothelial growth factor (VEGF-A) [90], which contributes to neo-angiogenesis.

Serum miR-199a-5p [84] and miR-370-3p [85] were downregulated in women with endometriosis, affecting cell proliferation. miR-370-3p targets SF-1, a gene regulator involved in estrogen biosynthesis; thus, the overexpression of SF-1 will increase local estrogen production [85]. The expression levels of miRNA may differ according to the time of the day when the blood sample is collected [91]. Thus, there is a challenge in the consistency of the studies investigating the expression levels of miRNAs. The heterogeneity of the patient population, the timing of the sample collection, and the menstrual cycle may be confounding factors in the inconsistency of these studies; thus, standardization in sampling design and bioinformatic assessments of miRNAs assays are required to ensure an improvement in identifying diagnostic markers involved in miRNAs for endometriosis.

## 4. Apoptosis in Endometriosis

### 4.1. Apoptosis

Apoptosis is a form of programmed cell death that eliminates cells from the tissue without evoking an inflammatory response [92]. The three phases of the programmed cell death cascade are signal activation triggering apoptosis, regulation and execution, and cellular structural modifications [92]. During the menstrual cycle, apoptosis is essential in regulating cellular homeostasis through the elimination of cells from the functional layer of the uterine endometrium, and it is detected in the glandular epithelium of the late secretory and menstruating phase [93,94]. Bcl-2 family and Fas/FasL are known to be involved in apoptosis in normal endometrium, with Bcl-2 exhibiting an inhibitory effect on apoptosis during the proliferative phase while Fas showed peak expression during the secretory phase in the menstrual cycle and initiate the caspase cascade by activating caspase-8 [95,96]. The activation will trigger the death pathway and cause apoptosis in the endometrium. Other genes in Bcl family are Bax and Bak which have a proapoptotic function which peaks during the secretory phase [97].

There is evidence of a reduced level of spontaneous apoptosis in the ectopic endometrium compared with the eutopic endometrium of women with endometriosis [93]. Furthermore, for the first time, Gebel et al., (1998) displayed a significant reduction of apoptosis in the eutopic endometrium of women with endometriosis compared to healthy controls [93]. Thus, it is hypothesized that the development of endometriotic lesions may be due to the reduced sensitivity of endometrial tissue to apoptosis.

### 4.2. microRNAs Regulate Apoptosis in Endometriosis

Ten studies from 2016 to 2022 on the dysregulated miRNAs that modulate apoptosis in endometriosis were found and listed in Table 4. These studies used different cell lines to represent endometriotic cell lines to study the effect of overexpression or knockdown of microRNAs on the target genes expressed. The miRNAs were downregulated, while the target genes were upregulated; these modifications may contribute to the antiapoptotic behavior of the endometrial stromal cells, thus causing the progression of endometriosis.

miR-2861, known to induce apoptosis, was found by Yu et al., (2019) to be significantly downregulated in ectopic endometrial tissues when compared to the eutopic endometrial tissue of healthy controls. miR-2861 markedly inhibited ectopic endometrial cell proliferation and induced apoptosis, implying its inhibitory effects on endometriosis [71]. This study confirmed that STAT3 and MMP2 were the targets of miR-2861 in cell proliferation and apoptosis [71]. For women with endometriosis, Yoo et al., (2016) found that the activation of STAT3 may be necessary for the development of the inflammatory phenotype of eutopic endometrium [98], and the aberrant activation of STAT3 signaling could contribute to the pathogenesis of endometriosis [99]. Thus, the downregulated miR-2861 may contribute to the upregulation of STAT3 and MMP2, thereby promoting cell proliferation and inhibiting apoptosis of the ectopic endometrial cells. STAT3 was found to be targeted by both miR-2861 [71] and miR-424-5p, and their downregulation could inhibit apoptosis in endometriosis.

**Table 4:** Studies on dysregulated miRNAs that modulate apoptosis in women with endometriosis.

Study	Sample Design	Age (Years)	Stages of Endometriosis (ASRM)	Dysregulated miRNAs	Target mRNA
Yu et al., (2019) [71]	EU & EC (*n* = 19) vs. C (*n* = 12)	39.3, 34-43	-	miR-2861	MMP2, STAT3
Hu et al., (2019) [85]	EU & EC (*n* = 12) vs. C (*n* = 10), serum (*n* = 46)	24-42	III and IV	miR-370-3p	SF-1, StAR, CYP19A1
Yang et al., (2017) [100]	EU & EC (*n* = 26) vs. C (*n* = 26)	30 ± 5.1		miR-424-5p	FGFR1, STAT3
Hirakawa et al., (2016) [101]	EC (*n* = 32) vs. C (*n* = 8)	EC (37-51), C (26-51)	-	miR-503	Cyclin D1, Rho A, Bcl-2, ROCK1, ROCK2, VEGF-A
Gao et. al. (2019) [102]	E (*n* = 40) vs. C (*n* = 20)		I, II, III and IV	miR-451	YWHAZ, OSR1, TTN, CDKN2D
Zhou et al., (2019) [103]	EC (*n* = 71) vs. C (*n* = 26), serum from both groups	E (31.84 ± 6.28), C(46.69 ± 4.15)	II and III	miR-205-5p	ANGPT2
Ma et al., (2019) [104]	EC (*n* = 20) vs. C (*n* = 20)	-	II-IV	miR-142-3p	KLF9, VEGFA
Zhang et al., (2019) [105]	EC	-		miR-141-3p	Kruppel factor 12 (KLF-12)
Rezk et al., (2021) [106]	EU & EC (*n* = 70) vs. C (*n* = 40)	-	I, II, III and IV	miR-34a	SIRT1/FoxO1/p53, Bax, Bcl-2, Bcl-xL
Tan et al., (2022) [107]	EC (*n* = 28) vs. C (*n* = 17)	EC (31.46 ± 6.20), C(32.76 ± 4.78)		miR-142-3p	CXCR7

Downregulation of miR-33 in ectopic endometriosis increases VEGF and matrix metalloprotein (MPP) expression and results in endometrial cell apoptosis [108]. MMP has been implicated in the conversion of TNF-α and FasL to active soluble forms, suggesting that these molecules can activate or release the factors involved in the apoptotic process [109]. In endometriotic lesions, the expression levels of MMP-2, MMP-9, and Mt1-MMP are significantly higher than a normal eutopic endometrium [110], implying that endometriotic tissue has a greater capacity for invasion. Thus, the higher expression of MMP9 and MMP2 from the previous studies may contribute to the inhibition of apoptosis of endometrial stromal cells in endometriosis. In the study of Gao et al., (2019), the eutopic endometrium of endometriosis patients and cell lines had considerably lower expression levels of miR-451 than the controls. The expression of miR-451 and the ASRM stage of endometriosis were not shown to be significantly correlated [102]. Cell growth is inhibited, and apoptosis is induced when miR-451 is overexpressed in endometriosis cell lines. Bioinformatic research of the GSE7846 dataset revealed the possible target genes for miR-451 to be YWHAZ, OSR1, TTN, and CDKN2D. Joshi et al., (2015) discovered the relationship between the expression of YWHAZ and miR-451 by encouraging the growth of ectopic cells in baboons with endometriosis [111]. In the study by Hirakawa et al., (2016), it was discovered that the hypermethylation of the miR-503 gene caused overexpression of this miRNA, which inhibited cell proliferation, VEGF-A production, extracellular matrix (ECM) contraction, and angiogenic activity of ECSCs, as well as induced apoptosis activity and G0/G1 cycle arrest of these cells. Since cyclin D1 was discovered to have a negative correlation with the expression of miR-503 and to inhibit cell proliferation, it was established that Cyclin D1, Bcl-2, and VEGF-A are direct targets of miR-503 in ECSC [101]. As an antiapoptotic factor with an elevated expression in ECSC, Bcl-2 may be a target for miR-503, which causes endometriosis cells to die by downregulating Bcl-2 expression. According to Ma et al., (2019), the overexpression of miR-142-3p inhibits the proliferation and migration of CRL-7566 cells and causes a decrease in miR-142-3p expression in ectopic endometrial tissues [104]. KLF9 is miR-142-target 3p’s gene, and this miRNA causes apoptosis via encouraging KLF9-mediated autophagy. By directly controlling the production of vascular endothelial growth factor A (VEGFA), KLF9 also encourages angiogenesis. The overexpression of miR-142-3p inhibits cell proliferation, as evidenced by a decrease in the proportion of cells in the S phase and an increase in the proportion of cells in the G1 phase in CRL-7566 and ECSC. MiR-142-3p controls endometrial cell invasion and migration, and it lowers VEGFA expression, which prevents vascularization. MiR-142-3p targets KLF9, and the overexpression of this gene in CRL-7566 cells promotes the expression of Bax and p62 while suppressing the expression of Bcl-2 and LC3B.

**Figure 2:**
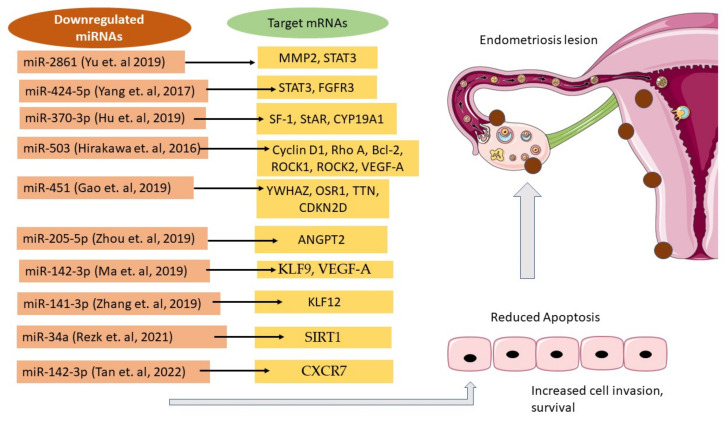
The list of downregulated miRNAs, and their target mRNAs involved in apoptosis in endometriosis [71,85,100,101,102,103,104,105,106,107].

As evidenced by Zhang et al., (2019), there is an increase in the expression of lentivirus miR-205-5p-transfected EC109 and EC520 endometriotic stromal cells relative to the negative controls, and miR-205-5p plays a suppressive role in endometriosis in vitro. Increased miR-205-5p expression decreases endometriotic stromal cells’ capacity for cell invasion and migration while increasing apoptosis [105]. Through the AKT/ERK signaling pathway, the ANGPT2 levels were significantly decreased in overexpressed miR-205-5p EC109 and EC520 cells [103]. This study also concentrated on the relationship between miR-205-5p downregulation and ANGPT-2 upregulation and the severity of endometriosis progression. Through a multivariate analysis, they discovered a significant correlation, demonstrating their promising potential as endometriosis biomarkers. The overexpression of Sirutin 1 (SIRT1) and Bcl-xL (antiapoptotic marker) in the ectopic and eutopic endometrial tissues of women with endometriosis compared with normal endometrial tissues may suggest their association with the progression of endometriosis, as studied by Rezk et al., (2021) [106]. This study found there is a reduced expression of miR-34a and Fox0-1 in the ectopic and eutopic endometrial samples. This implies that miR-34a may play a role in regulating the expression of SIRT1 by the p53 pathway and subsequently regulating FoxO-1 expression in endometriosis tissue by decreasing apoptosis in the endometrium. Downregulated miRNAs that link to cell apoptosis in endometriosis are illustrated in Figure 2.

### 4.3. Abnormal microRNA Expression and Failed Decidualization and Their Association with Apoptosis in Endometriosis

Infertility or failed embryo implantation is one of the most prominent symptoms of endometriosis. According to Hu et al., (2018), endometriosis patients’ serum samples had significantly lower expression levels of miR-370-3p compared to the controls. StAR, CYP19A1, and SF 1 downstream gene expression were shown to be regulated by miR-370-3p. According to the study, endometriotic cells that have miR-370-3p overexpression undergo apoptosis and have decreased cell growth. The endometrium’s ability to decidualize, which results in infertility, is inhibited by SF-1, which also encourages aberrant uterine gland morphogenesis, changes the endometrial immune homeostasis, and causes a physiological inflammatory response [85].

This review has its strengths. The findings and discussion about the miRNAs and their target mRNAs that regulate cell apoptosis in endometriosis are comprehensive and robust. We also highlighted the circulating miRNAs that may give insight into the diagnosis and management of endometriosis. However, our review also has two limitations. First, we could not find suitable circulating miRNA biomarkers or common miRNAs that link to cell apoptosis in endometriosis. Second, the limited English full articles might contribute to the limitations of our findings.

## 5. Conclusions

microRNAs regulate various biological pathways involved in endometriosis by repressing the translation of the target mRNAs. They play a role in inflammatory and immune responses, cell adhesion, cell invasion, cell proliferation, angiogenesis, apoptosis, cell differentiation, and hormone synthesis. There are pieces of evidence of miRNAs in regulating the development and progression of endometriosis, which is important for future directions in the treatment of the disease. Many studies show different expression profiles of miRNA in endometrium tissue and endometrial lesions. A microRNA expression profile may therefore play a diagnostic role, as well as a prognostic factor for the efficacy of novel therapies.

In conclusion, the significance of miRNAs in the regulation of the development of endometriosis is well-known currently, but there are still many questions left to be answered. New research techniques and functional studies on the role of miRNA and their target mRNAs will be needed to improve our knowledge of the pathophysiological impact of these miRNAs in endometriosis. This will enable a more effective treatment and potential diagnostic biomarkers to be used as the future therapeutic targets for the disease.

## Figures and Tables

**Table 1:** Studies demonstrating altered expression in ectopic vs. eutopic endometrial tissue of women with endometriosis.

Study	Sample Design	Age (Years)	Location of Lesion	Menstrual Cycle (in Endometriosis)	Dysregulated miRNAs
Filigheddu et al., (2010) [21]	Paired sample of eutopic (EU) vs. ectopic lesion (EC) (*n* = 3)	24 to 48 (mean 36)	Ovarian (*n* = 3)	Early proliferative	Upregulated: miR-1, 100, 101, 126, 130a, 143, 145, 148a, 150, 186, 199a, 202, 221, 28, 299-5p, 29b, 29c, 30e-3p, 30e-5p, 34a, 365, 368, 376a, 379, 411, 493-5p and 99a Downregulated: miR-106a, 106b, 130b, 132, 17-5p, 182, 183, 196b, 200a, 200b, 200c, 20a, 25, 375, 425-5p, 486, 503, 638, 663, 671, 768-3p, 768-5p, and 93
Ramon et al., (2011) [48]	Paired eutopic endometrium vs. ectopic endometrium (*n* = 41), ovarian endometrioma (*n* = 41) vs. eutopic controls (*n* = 38)	Endometriosis (24–47, mean 34.9) vs. control (21–47, mean 36.3)	Ovarian (*n* = 41), peritoneal (*n* = 24) and rectovaginal (*n* = 13)	Proliferative (*n* = 26) secretory (*n* = 32)	Upregulated: miR-21, miR-125 and miR-222 Downregulated: miR-15b, miR-17-5p and miR-20a
Braza-Boils et al., (2014) [66]	Paired ovarian endometriomas vs. eutopic endometrial tissue (*n* = 51) (peritoneal implants 18/51, rectovaginal nodules 20/51) vs. control (*n* = 32)	Endometriosis (20–45, mean 35) Control (27–45, mean 36.4)	Ovarian (*n* = 51), peritoneal (*n* = 18/51), rectovaginal nodules (*n* = 20/51)	Proliferative (*n* = 26), Secretory (*n* = 25)	Upregulated: miR-29c-3p, -138, -202-3p, -411-5p, -411-3p, -424-5p Downregulated: miR-16, -373-3p, -449b-3p,-556-3p,-636,-935
Rekker et al., (2018) [67]	Paired eutopic endometrium (*n* = 4) vs. ectopic endometrium (*n* = 4)	Mean 32.0 years	Unspecified	Proliferative	Upregulated: miR-139-5p, -139-3p, -202-5p, -506-3p, -150-5p, -202-3p, -150-3p, -513c-5p, -193a-5p, -584-5p, -371a-5p, and 216b-5p Downregulated: miR-375, -105-5p, -1298-5p, -6507-5p, -767-5p, -675-3p, -429, -141-3p and -873-5p
Shen et al., (2013) [68]	Eutopic and ectopic endometrial tissue (*n* = 23)	20–44 years	Unspecified	Proliferative (*n* = 23)	Downregulated: miR-23a and miR-23b
Shi et al., (2014) [69]	Paired eutopic vs. ectopic endometriosis (*n* = 20) vs. controls (*n* = 20)	Normal Mean 38.9 years vs. endometriosis (36.6 years)	Ovarian	Proliferative	Upregulated: miRPlus-F1038, -1915, -637, -518e, -519a, -519b-5p, -519c-5p, -522, -523, -574-5p, -615-3p, -1909, -224, -133b, -622, -628-3p, miRPlus-F1215, miRPlus-F1221, -1470, -1469, 520d-5p, -551b, -361-3p, -941 Downregulated: miR-203, -425, -183, -92a, -196b, -215, -363, let-7i, -miRPlus-E1031, -200b, miRPlus-F1231, -362-3p, -342-3p, -200c, -93, -24-1, -25, -106b
Wang et al., (2013) [70]	Eutopic vs. ectopic endometriotic lesion (*n* = 19) vs. controls (*n* = 12)	34–43 (mean 39.3 years)	Unspecified	Proliferative	Downregulated: miR-195
Yu et al., (2019) [71]	Paired eutopic and ectopic endometriotic lesion (*n* = 19)	34–43 (mean 39.3 years)	Unspecified	Unspecified	Downregulated: miR-2861

EU: eutopic endometrium and EC: ectopic tissue.

**Table 2:** Studies demonstrating the altered expression of miRNAs in eutopic endometrium of women with or without endometriosis.

Study	Sample Design	Age	Location of Lesion	Menstrual Cycle (In Endometriosis)	Dysregulated miRNAs
Burney et al., (2009) [45]	Eutopic endometrium of endometriosis (*n* = 4) vs. eutopic controls (*n* = 3)	23–50 years	Ovarian, peritoneal and rectovaginal nodules	Early secretory	Downregulated: miR-9, miR-9*, miR-34b*, miR-34c-5p, miR-34c-3p and miRPlus_42 780.
Aghajanova and Giudice (2011) [75]	Eutopic severe endometriosis (*n* = 44) vs. eutopic mild endometriosis (*n* = 19)	20–48 (mean 35)	Unspecified	Early to mid-secretory	Upregulated miR-21 in severe vs. mild endometriosis throughout the menstrual cycle
Pei et al., (2018) [76]	Eutopic endometrium of endometriosis (*n* = 19) vs. controls (*n* = 14)	20–35 years	Unspecified	Mid-secretory	Upregulated: miR-194-3p
Liu et al., (2018) [77]	Ectopic (endometrioma; *n* = 19), eutopic (*n* = 19), and normal (*n* = 35) endometrial tissues from patients with or without endometriosis	normal group were 32.7 ± 6.8 years, and that of the endometriosis group was 34.6 ± 5.2 years.	Unspecified	Proliferative phase	Downregulated: miR-449b-3p in ectopic and eutopic tissues from women with endometriosis.
Yang et al., (2019) [78]	Eutopic endometrium of endometriosis (*n* = 38) vs. controls (*n* = 38)	20–40 years	Unspecified	Proliferative	Upregulated: miR-142-5p, miR-146a-5p, miR-1281, miR-940, and miR-4634 Downregulated miR-543
Zhou et al., (2016) [79]	Eutopic endometrium from women with endometriosis (*n* = 20) vs. controls eutopic (*n* = 20)	20–35 years	Unspecified	Mid-secretory	54 miRNAs downregulated, 12 upregulated Upregulated: miR-196a overexpression inhibited PGR and PGR-B mRNA expression, whereas inhibition of miR-196a elevated PGR and PGR-B mRNA expression.

**Table 3:** Studies demonstrating differences in circulating miRNAs in women with endometriosis.

Study	Sample Design	Age	Location of Lesion	Menstrual Cycle	Dysregulated miRNAs
Cho et al., (2015) [83]	Serum miRNA expression in women with endometriosis (*n* = 24) vs. control (*n* = 24)	18–48 years	Ovarian (*n* = 24) (100%), coexisting peritoneal (*n* = 22) and coexisting deep infiltrating endometriosis (*n* = 8)	Proliferative, secretory	Downregulated Let-7b, let-7d, let-7f and miR-135a
Hsu et al., (2014) [84]	Serum from women with endometriosis (*n* = 40) vs. control (*n* = 25)	18–53 years	Ovarian (*n* = 36), peritoneal (*n* = 4)	Unspecified	Downregulated: miR-199a-5p
Hu et al., (2019) [85]	Serum from women with endometriosis (*n* = 20) vs. women without the disease (*n* = 26)	18–49 years	Ovarian	Proliferative	Downregulated miR-370-3p
Jia et al., (2013) [86]	Plasma miRNA from women with endometriosis (*n* = 3) vs. without the disease (*n* = 3). Plasma sample for validation of miRNA profiling from women with endometriosis (*n* = 20) and control (*n* = 20)	25–44 years (mean 34)	Unspecified	Proliferative, secretory	Downregulated: miR-122, 17-5p, 19, 20a, 19b, 15b-5p, 451, 30e, 27a, 92, 26a, 320e, 320d, 762, 1274b, 630, 29c, 223, 22, 1268, 23a, H10, 21, 3679, 30d, 572 and 3665
Maged et al., (2018) [87]	Blood and peritoneal fluid from women with endometriosis (*n* = 45) vs. control (*n* = 35)	Endometriosis (mean 29.64) vs. control (mean 29.46). maximum age of 37 years	Pelvic cavity	Proliferative phase	Upregulated miR-199a and 122
Nematian et al., (2018) [88]	Serum from women with endometriosis (*n* = 20) vs. without disease (*n* = 26)	18–49 years			Upregulated miR-125b and downregulated Let-7

## Data Availability

Not applicable.
